# Reduced Verbal Fluency following Subthalamic Deep Brain Stimulation: A Frontal-Related Cognitive Deficit?

**DOI:** 10.1371/journal.pone.0140083

**Published:** 2015-10-08

**Authors:** Jean-François Houvenaghel, Florence Le Jeune, Thibaut Dondaine, Aurore Esquevin, Gabriel Hadrien Robert, Julie Péron, Claire Haegelen, Sophie Drapier, Pierre Jannin, Clément Lozachmeur, Soizic Argaud, Joan Duprez, Dominique Drapier, Marc Vérin, Paul Sauleau

**Affiliations:** 1 “Behaviour and Basal Ganglia” research unit (EA 4712), University of Rennes, Rennes, France; 2 Department of Neurology, Rennes University Hospital, Rennes, France; 3 Department of Nuclear Medicine, Eugène Marquis Hospital, Rennes, France; 4 Department of Neuroradiology, Rennes University Hospital, Rennes, France; 5 Department of Psychiatry, Rennes University Hospital, Rennes, France; 6 ‘Neuroscience of Emotion and Affective Dynamics’ laboratory, Department of Psychology, and Swiss Center for Affective Sciences, University of Geneva, Geneva, Switzerland; 7 Department of Neurosurgery, Rennes University Hospital, Rennes, France; 8 “MediCIS” laboratory (UMR 1099 LTSI), Inserm/University of Rennes, Rennes, France; 9 Department of Neurophysiology, Rennes University Hospital, Rennes, France; Oslo University Hospital, NORWAY

## Abstract

**Objective:**

The decrease in verbal fluency in patients with Parkinson’s disease (PD) undergoing subthalamic nucleus deep brain stimulation (STN-DBS) is usually assumed to reflect a frontal lobe-related cognitive dysfunction, although evidence for this is lacking.

**Methods:**

To explore its underlying mechanisms, we combined neuropsychological, psychiatric and motor assessments with an examination of brain metabolism using F-18 fluorodeoxyglucose positron emission tomography, in 26 patients with PD, 3 months before and after surgery. We divided these patients into two groups, depending on whether or not they exhibited a postoperative deterioration in either phonemic (10 patients) or semantic (8 patients) fluency. We then compared the STN-DBS groups with and without verbal deterioration on changes in clinical measures and brain metabolism.

**Results:**

We did not find any neuropsychological change supporting the presence of an executive dysfunction in patients with a deficit in either phonemic or semantic fluency. Similarly, a comparison of patients with or without impaired fluency on brain metabolism failed to highlight any frontal areas involved in cognitive functions. However, greater changes in cognitive slowdown and apathy were observed in patients with a postoperative decrease in verbal fluency.

**Conclusions:**

These results suggest that frontal lobe-related cognitive dysfunction could play only a minor role in the postoperative impairment of phonemic or semantic fluency, and that cognitive slowdown and apathy could have a more decisive influence. Furthermore, the phonemic and semantic impairments appeared to result from the disturbance of distinct mechanisms.

## Introduction

A moderate decline in verbal fluency is the most frequent neuropsychological side effect of deep brain stimulation of the subthalamic nucleus (STN-DBS) in Parkinson’s disease (PD) [1,2], and seems to concern 30-40% of patients [3]. As verbal fluency is regarded as an executive function that draws on many other executive processes, including word retrieval, verbal working memory, inhibition and flexibility, its postoperative decline is usually assumed to reflect dysfunction in frontal lobe-related cognitive functions [4–7]. This hypothesis is, in part, supported by three studies that have investigated the relationship between phonemic [8,9] or semantic [10] fluency and brain activity following STN-DBS surgery in patients with PD. The decline in performance has mainly been associated with decreased activity in the frontal [9], frontotemporal [8] or frontostriatal networks [10], including areas known to be involved in word retrieval [8,9] and response inhibition [8–10], supporting the hypothesis of a deficit in executive functions underlain by frontal areas [8–10]. Nevertheless, this hypothesis can be challenged for at least two reasons. First, these studies did not demonstrate a possible relationship between a decrease in verbal fluency and changes in other frontal-related cognitive functions such as inhibition. Second, owing to the absence of a relationship between postoperative impairment in verbal fluency and other executive functions assessed using neuropsychological tests [11–16], some authors have suggested that this deficit is the consequence not of a specific executive dysfunction, but of an overall and nonspecific cognitive slowdown following surgery [17,18]. In addition, it has been suggested that other behavioural changes following surgery, such as increased apathy, also play a role [14,19].

To explore the neural and behavioural domains related to verbal fluency deterioration, we prospectively assessed the relationship between postoperative verbal fluency deficits and changes in brain metabolism and neuropsychological, psychiatric or motor measures. We investigated the neural substrates of verbal fluency deficits following STN-DBS in a large cohort of 26 patients with PD using 2-deoxy-2[18F]fluoro-D-glucose (^18^F-FDG) positron emission tomography (PET), performed 3 months before and after STN-DBS surgery. We then split the cohort into two groups, according to the presence or absence of a postoperative decline in verbal fluency, as the aim of the present study was to determine the changes that specifically occur in patients with a postoperative decline. For this purpose, and to take disease progression and the test-retest effect into consideration, we used a group of 34 patients with PD who did not undergo STN-DBS, whom we matched at baseline with the STN-DBS group. We then compared the two STN-DBS groups with and without impaired verbal fluency on the basis of metabolic, neuropsychological, psychiatric and motor changes. Furthermore, as verbal fluency is classically divided in phonemic and semantic forms, we studied changes in these two components.

## Methods

### Patients

Participants were 26 patients (13 men and 13 women) with idiopathic PD [20] who had been selected for bilateral STN-DBS (Table 1). Standard selection and exclusion criteria for STN-DBS were applied to all patients [21]. Mean (± *SD*) age at surgery was 56.6 (± 7.4) years, education level was 10.6 (± 2.8) years, and disease duration at surgery was 11.5 (± 4.5) years. Patients with significant vascular abnormalities and brain atrophy were excluded on the basis of preoperative MRI scans. Those with dementia or major depression were also excluded, on the basis of preoperative neuropsychological and psychiatric assessments (Mattis Dementia Rating Scale, MDRS; Montgomery-Åsberg Depression Rating Scale, MADRS) [22,23]. All patients were right-handed, according to the Edinburgh Handedness Inventory [24].

**Table 1:** Clinical assessment (mean ± *SD*) of patients with PD before (preoperative condition) and after (postoperative condition) STN-DBS surgery.

	On-dopa condition		Off-dopa condition	
	Preoperative (M-3)	Postoperative (M+3)	Preoperative (M-3)	Postoperative (M+3)
	Mean ± *SD*	Mean ± *SD*	Mean ± *SD*	Mean ± *SD*
UPDRS-III	**8.3 ± 5.7**	**5.0 ± 3.8 ** ***	**31.4 ± 11.5**	**14.5 ± 9.4 ** ***
Schwab & England (%)	**86.9 ± 9.7**	94.2 ± 5.8 ***	**66.1 ± 21.2**	78.8 ± 13.4 **
Hoehn & Yahr	** 1.0 ± 0.8**	0.7 ± 0.8 *	** 2.4 ± 0.9**	1.5 ± 0.9 ***
MDRS	140.4 ± 3.0	139.8 ± 3.7		
Verbal fluency				
Semantic	30.1 ± 10.6	29.0 ± 10.1		
Phonemic	**22.0 ± 7.2**	18.8 ± 6.9***		
Stroop				
Colour	71.8 ± 14.4	67.9 ± 11.9		
Word	101.5 ± 18.4	99.0 ± 16.7		
Colour-Word	42.6 ± 10.8	41.7 ± 8.3		
Interference	0.9 ± 7.1	1.4 ± 7.6		
TMT				
Part A	45.7 ± 16.1	45.2 ± 14.5		
Part B	106.0 ± 53.2	125.6 ± 82.3		
B-A	60.3 ± 44.7	80.3 ± 75.3		
MCST				
Categories	5.5 ± 0.9	5.8 ± 0.3		
Errors	5.4 ± 5.0	4.1 ± 3.6		
Perseverations	1.4 ± 1.8	1.4 ± 1.7		
MADRS	5.6 ± 4.9	4.0 ± 3.8		
AES	31.8 ± 7.0	31.2 ± 7.7		

*Note*. Statistical effects between the two conditions (pre- and post-surgery) are reported (Wilcoxon’s test for paired comparisons).

* *p* < 0.05

** *p* < 0.01

*** *p* < 0.005

Postoperative assessments were performed with the stimulator ON.

*Abbreviations*. *SD* = standard deviation; UPDRS-III = Unified Parkinson’s Disease Rating Scale Part III; TMT: Trail Making Test; MCST = Modified Wisconsin Card Sorting Test; MADRS = Montgomery and Asberg Depression Rating Scale; MDRS = Mattis Dementia Rating Scale; AES = Apathy Evaluation Scale.

Each of the patients underwent motor, psychiatric, neuropsychological and PET assessments within the same week, 3.1 (± 2.4) months before and 3.1 (± 0.6) months after surgery. All the patients were on dopaminergic medication (ON medication) preoperatively, and ON medication and ON stimulation for all postoperative assessments. Patients underwent the neuropsychological tests with dopaminergic medication to limit the effect of motor symptoms on cognitive performances, and because ON medication better reflected the patients’ clinical status in everyday life. Verbal fluency deterioration has been shown to be an early and stable cognitive side effect that occurs as early as the third month and remains stable between 3 and 12 months [25].

To control for disease duration and the test-retest effect with a 6-month interval between verbal fluency performances, we recruited a group of 34 patients with PD who underwent the same assessments as the STN group (except for the PET scan) twice, 6 months apart. The same exclusion criteria were applied as for the STN group. This group was comparable to the STN group on sex (17 men), age (56.6 ± 10.8 years), education level (11.1 ± 4.1 years), disease duration (9.9 ± 6.2 years), medication (1164.8 ± 389.0 mg levodopa equivalent daily dose) and all baseline motor, psychiatric and neuropsychological scores (*p* > 0.05 for each comparison).

After providing a complete description of the study, we obtained written informed consent from each participant. The study was approved by the local ethics committee of University of Rennes and conducted in accordance with the Declaration of Helsinki.

### Motor assessment

All participants were assessed in accordance with the Core Assessment Program for Intracerebral Transplantation [26], and their disease severity was rated on the Unified Parkinson’s Disease Rating Scale (UPDRS), and the Hoehn and Yahr and Schwab and England scales.

### Neuropsychological assessment

We administered phonemic (letter *p*) and semantic (animals) verbal fluency tasks to all the participants [27]. We recorded the numbers of words produced within two minutes, excluding repetitions and intrusions. To assess other executive functions, we used a neuropsychological battery that included Nelson’s simplified version of the Wisconsin Card Sorting Test (MCST), the Trail Making Test (TMT) and the Stroop Interference Test [28–30].

### Psychiatric assessment

Apathy was assessed by an experienced psychiatrist using the Apathy Evaluation Scale (AES) [31]. This scale is composed of four subscales: behaviour (impairment in initiating and sustaining goal-directed behaviour), cognition (decrease in goal-related thought content), emotion (decrease in emotional responses following goal-related events) and other (initiative and motivation).

### Neurosurgery and stimulation settings

Surgery was performed under local anaesthesia, using MRI determination of the target, microrecording and intraoperative assessment of the clinical effects induced by stimulation. The correct position of the electrodes was checked postoperatively, using a 3D CT brain scan. Quadripolar electrodes (3389, Medtronic, Minneapolis, MN, USA) were implanted bilaterally in all the patients. Three months after surgery, the mean coordinates of the selected contacts were 13.5 ± 2.1 mm lateral to, 2.4 ± 2.0 mm below, and 2.0 ± 2.0 mm posterior to the midpoint of the bicommissural (AC-PC) line on the right side, and 13.5 ± 1.6 mm lateral to, 3.1 ± 1.5 mm below, and 2.2 ± 1.9 mm posterior to the midpoint of the AC-PC line on the left side. The mean parameters for the monopolar stimulation were 2.2 (± 0.5) volts, 61.1 (± 5.9) μs and 131.3 (± 5.9) Hz on the right side, and 2.0 (± 0.6) volts, 61.2 (± 5.9) µs and 130.2 (± 1.0) Hz on the left side.

### PET imaging procedure and image transformation

#### PET imaging procedure

Patients were night fasted for the PET scans. There was no statistical difference in fasting serum glucose levels at the time of the PET measurements before and after implantation. The scans were performed using a dedicated Discovery ST PET scanner (GEMS, Milwaukee, MN, USA) in 2D mode, with an axial field of view (FOV) of 15.2 cm. A 222-296 MBq injection of ^18^F-FDG was administered intravenously in a resting state. A 20-minute 2D emission scan was performed 30 minutes post injection, after X-ray based attenuation correction. Following scatter, deadtime and random corrections, PET images were reconstructed using 2D filtered back-projection. This yielded 47 contiguous, transaxial 3.75-mm thick slices.

#### PET image transformation

For the present study, we used a method that has already been described elsewhere [32]. The data were analysed with statistical parametric mapping (SPM8; Wellcome Department of Cognitive Neurology, London, UK) implemented in MATLAB Version 2010b (Mathworks Inc., Sherborn, MA, USA). Statistical parametric maps are spatially extended statistical processes used to characterize regionally specific effects in imaging data. SPM combines the general linear model (to create the statistical map) and the theory of Gaussian fields to make statistical inferences about regional effects [33]. The effect of overall differences in blood flow was removed using proportional scaling, with the global mean set at 50 and the masking threshold set at 0.8. No other preprocessing was carried out.

Images were first realigned and spatially normalized into standard stereotactic space according to the MNI atlas. Affine transformation was performed to determine the 12 optimum parameters for registering the brain to the template. The subtle differences between the transformed image and the template were then removed using a nonlinear registration method. Finally, the spatially normalized images were smoothed using an isotropic 12-mm full width at half-maximum isotropic Gaussian kernel to compensate for interindividual anatomical variability and to render the imaging data more normally distributed.

### Statistical analysis

#### Clinical assessment

As phonemic and semantic performances were normally distributed, we were able to calculate a reliable change index (RCI) for each patient. This technique allowed us to distinguish between patients with and without a decrease in verbal fluency following STN-DBS, taking disease progression and the test-retest effect into consideration [34]. The RCI was calculated with the following formula: ((*X*2—*X*1)—(*M*2—*M*1)) / SDD, where *X*1 and *X*2 were the baseline and follow-up scores for each patient, *M*1 and *M*2 the baseline and follow-up means of the 40 patients with PD without STN-DBS and SDD the standard deviation of the difference between the follow-up minus baseline scores in the group without DBS. Patients with a low RCI score (≤ -0.5) were assigned to the group with a verbal fluency deficit, and patients with an RCI > -0.5 to the group without a verbal fluency deficit. This index was further used to define four subgroups: patients with or without a decrease following STN-DBS for phonemic verbal fluency and for semantic verbal fluency. Using the Mann-Whitney *U* test, we then compared the stimulation parameters of the groups with and without impaired fluency, as well as changes in dopaminergic treatment, and motor, psychiatric and neuropsychological scores following STN-DBS. The same comparisons were carried out for each type of verbal fluency. To complete the analyses, we looked for correlations between verbal fluency changes and other behavioural changes using Spearman's rho test. The significance threshold was set at *p* = 0.05 for all analyses.

#### Brain metabolism analyses

SPM software was used to calculate significant changes in brain metabolism in the groups with and without phonemic or semantic verbal fluency. We ran a 2 (group: with or without postoperative deterioration) x 2 (phase: pre- or postoperative PET imaging) analysis of variance (ANOVA). No covariate was included in the statistical model.

Clusters that survived a two-tailed threshold of *p* < 0.001, with multiple-comparison correction, and corrected at the cluster level (*p* < 0.05), were deemed to be significant. All the coordinates reported here were based on the MNI atlas.

## Results

### Clinical assessments

Considering the whole cohort, the mean coordinates of the selected contacts and the major motor benefit following surgery confirmed the correct positioning of the active contacts within the STN (Table 1).

Based on the RCI, 10 patients were included in the subgroup with phonemic deterioration (38%) and 16 in the subgroup without phonemic deterioration, while 8 were included in the subgroup with semantic deterioration (31%) and 18 in the subgroup without semantic deterioration. Only two patients displayed deterioration in both phonemic and semantic fluency. Stimulation parameters did not differ between the phonemic and semantic fluency subgroups.

Compared with the patients with no phonemic fluency impairment, patients with impaired phonemic fluency exhibited a greater motor improvement, as indicated by a decrease in the UPDRS Part III score OFF medication with the stimulator ON (*p* = 0.009), but a lower reading speed, as revealed by the Stroop word score (*p* = 0.03) (Table 2). No other change following STN-DBS or preoperative difference was observed between these subgroups. In addition, the decrease in phonemic fluency was significantly correlated with the decrease in reading speed, as measured with the Stroop word score (*r* = 0.44, *p* = 0.03), and an increase in the time taken to complete the TMT A (*r* = -0.40, *p* = 0.05). No other change in behavioural scores (motor, psychiatric, neuropsychological), medication or demographic data (age, education, disease duration) correlated with the change in phonemic fluency.

**Table 2:** Comparisons of the clinical assessments (mean ± *SD*) at baseline (preoperative condition) and follow-up (postoperative condition), and the follow-up minus baseline (post-pre difference) difference between the PD groups with and without phonemic deterioration following STN-DBS.

	Preoperative (M-3)			Postoperative (M+3)		Post-pre difference		
	With deterioration	Without deterioration	*p* value	With deterioration	Without deterioration	With deterioration	Without deterioration	*p* value
Men/Women, (*N*)	5/5	8/8	1.00^a^					
Age (years)	58.0 ± 9.2	55.8 ± 6.2	0.24					
Education (years)	11.3 ± 3.4	10.1 ± 2.4	0.34					
Disease duration (years)	11.1 ± 5.4	11.7 ± 4.0	0.56					
UPDRS-III ON	8.8 ± 5.9	8.0 ± 5.8	0.54	3.9 ± 1.9	5.7 ± 4.5	-4.9 ± 6.4	-2.3 ± 4.0	0.32
UPDRS-III OFF	34.6 ± 13.8	28.3 ± 9.7	0.22	10.4 ± 5.5	17.0 ± 10.6	**-24.2 ± 12.7**	**-12.3 ± 6.8**	**0.009**
Speech item ON	0.3 ± 0.7	0.3 ± 0.4	0.80	0.1 ± 0.2	0.2 ± 0.3	-0.2 ± 0.6	-0.2 ± 0.5	0.79
Speech item OFF	1.2 ± 0.9	1.0 ± 0.7	0.50	0.4 ± 0.5	0.5 ± 0.6	-0.8 ± 0.9	-0.4 ± 0.8	0.28
Schwab & England (%) ON	86.0 ± 11.7	87.5 ± 8.6	0.89	94.0 ± 5.2	94.4 ± 6.3	8.0 ± 12.3	6.9 ± 9.5	0.89
Schwab & England (%) OFF	58.0 ± 25.7	71.2 ± 16.7	0.17	74.0 ± 9.7	81.9 ± 14.7	16.0 ± 25.0	10.6 ± 21.1	0.67
Hoehn & Yahr ON	1.2 ± 1.0	0.9 ± 0.7	0.28	1.0 ± 0.8	0.5 ± 0.8	-0.2 ± 0.9	-0.3 ± 0.6	0.84
Hoehn & Yahr OFF	2.6 ± 1.2	2.3 ± 0.7	0.54	1.8 ± 0.6	1.3 ± 1.0	-0.7 ± 1.2	-1.0 ± 0.9	0.39
LEDD (mg)	1176.0 ± 464.2	1330.7 ± 613.2	0.67	658.6 ± 336.6	820.1 ± 451.4	-517.4 ± 400.0	-510.6 ± 368.1	0.87
MADRS	6.2 ± 3.5	5.2 ± 5.7	0.38	4.1 ± 3.3	3.7 ± 4.2	-2.1 ± 4.2	-1.4 ± 4.9	0.45
AES	30.8 ± 5.5	32.4 ± 7.9	0.62	29.9 ± 5.8	32.0 ± 8.8	-0.9 ± 4.4	-0.4 ± 5.6	0.49
Cognitive items	13.1 ± 2.0	14.6 ± 3.5	0.25	13.0 ± 2.5	14.8 ± 4.0	-0.1 ± 2.0	0.2 ± 2.9	0.29
Behavioural items	8.3 ± 1.9	8.4 ± 2.1	0.79	8.5 ± 2.0	8.1 ± 2.3	0.2 ± 2.2	-0.2 ± 2.7	0.64
Emotional items	4.1 ± 0.9	4.2 ± 1.0	1.00	3.4 ± 1.0	3.7 ± 1.2	-0.7 ± 1.3	-0.4 ± 0.9	0.60
Other items	5.3 ± 1.6	5.3 ± 2.0	0.96	5.0 ± 1.6	5.3 ± 1.8	-0.3 ± 1.3	0.0 ± 1.4	0.65
MDRS								
Without verbal initiation	112.4 ± 1.5	111.2 ± 2.1	0.15	111.7 ± 1.4	111.1 ± 2.5	-0.7 ± 2.1	-0.1 ± 2.9	0.29
Verbal fluency								
Semantic	29.2 ± 12.3	30.6 ± 9.9	0.67	28.2 ± 10.7	29.6 ± 9.9	-1.0 ± 6.8	-1.1 ± 8.5	0.87
Phonemic	24.5 ± 6.4	20.5 ± 7.5	0.13	17.1 ± 7.6	19.9 ± 6.2	**-7.4 ± 2.7**	**-0.6 ± 2.6**	**<0.0001**
Stroop								
Colour	68.9 ± 14.1	73.7 ± 14.8	0.54	65.4 ± 16.1	69.6 ± 8.4	-3.5 ± 7.3	-4.1 ± 11.5	0.80
Word	98.3 ± 17.0	103.6 ± 19.6	0.64	90.7 ± 17.5	106.0 ± 13.5	**-7.6 ± 7.9**	**2.2 ± 11.8**	**0.03**
Colour-Word	44.0 ± 7.6	41.6 ± 12.7	0.89	43.6 ± 6.6	40.4 ± 9.3	-0.4 ± 4.7	-1.2 ± 8.7	0.80
Interference	3.6 ± 4.7	-0.9 ± 7.9	0.16	5.7 ± 6.2	-1.4 ± 7.3	2.1 ± 4.7	-0.5 ± 7.6	0.44
TMT								
Part A	48.4 ± 15.0	44.0 ± 16.9	0.38	51.8 ± 16.8	41.1 ± 11.6	3.4 ± 8.7	-2.9 ± 10.4	0.21
Part B	108.7 ± 50.2	104.2 ± 56.6	0.81	136.9 ± 81.9	118.5 ± 84.3	28.2 ± 48.2	14.2 ± 39.2	0.41
B-A	60.3 ± 44.2	60.2 ± 46.5	0.96	85.1 ± 76.9	77.4 ± 76.7	24.8 ± 50.9	17.1 ± 43.7	0.56
MCST								
Categories	5.7 ± 0.5	5.4 ± 1.0	0.63	5.7 ± 0.4	5.9 ± 0.2	0.0 ± 0.7	0.5 ± 1.0	0.37
Errors	5.5 ± 5.4	5.4 ± 4.9	0.82	4.5 ± 4.1	3.8 ± 3.3	-1.0 ± 5.5	-1.6 ± 4.4	0.63
Perseverations	1.6 ± 2.3	1.3 ± 1.5	0.95	1.2 ± 2.0	1.5 ± 1.5	-0.4 ± 2.9	0.5 ± 1.0	0.50

*Abbreviations*. *SD* = standard deviation; UPDRS-III = Unified Parkinson’s Disease Rating Scale Part III; LEDD = levodopa equivalent daily dose; MADRS = Montgomery and Asberg Depression Rating Scale; AES = Apathy Evaluation Scale; MDRS = Mattis Dementia Rating Scale; TMT = Trail Making Test; MCST = Modified Wisconsin Card Sorting Test.

^a^Compared with Fisher’s exact test.

Compared with the patients with no semantic fluency impairment, patients with a semantic fluency impairment displayed a greater postoperative improvement in speech, as shown by the decrease in the ON medication Speech subscore (Item 18) of the UPDRS Part III (*p* = 0.03), and a higher level of apathy, as assessed by the total AES score (*p* = 0.01) and, more specifically, by the AES Cognitive (*p* = 0.007) and Behavioural (*p* = 0.009) subscores (Table 3). The two subgroups also differed on two preoperative parameters. First, patients with a postoperative semantic fluency impairment had better preoperative semantic fluency performances than patients without impairment (*p* = 0.01), whereas there was no difference postoperatively (*p* = 0.52). Second, the subgroup with no postoperative deterioration had a lower preoperative Schwab and England score in the ON medication condition (*p* = 0.01). By contrast, this subgroup exhibited a greater postoperative increase in the Schwab and England ON medication score (*p* = 0.05) than the subgroup with impaired fluency. Further analyses confirmed the existence of a negative correlation between the decrease in semantic fluency and the increase in the total AES score (*r* = -0.46, *p* = 0.02), AES Cognitive score (*r* = -0.51, *p* = 0.008) and AES Behavioural score (*r* = -0.51, *p* = 0.008). No other change in behavioural scores (motor, psychiatric, neuropsychological), medication or demographic data (age, education, disease duration) correlated with the change in semantic fluency.

**Table 3:** Comparisons of the clinical assessments (mean ± *SD*) at baseline (preoperative condition) and follow-up (postoperative condition), and the follow-up minus baseline difference (post-pre difference) between the PD groups with and without semantic deterioration following STN-DBS.

	Preoperative (M-3)			Postoperative (M+3)		Post-pre difference		
	With deterioration	Without deterioration	*p* value	With deterioration	Without deterioration	With deterioration	Without deterioration	*p* value
Men/Women (*N*)	4/4	9/9	1.00^a^					
Age (years)	55.8 ± 6.6	57.0 ± 7.8	0.60					
Education (years)	11.9 ± 3.9	10.0 ± 2.1	0.25					
Disease duration (years)	11.4 ± 3.5	11.6 ± 4.9	0.89					
UPDRS-III ON	9.9 ± 6.1	7.6 ± 5.6	0.30	6.6 ± 5.0	4.3 ± 3.0	-3.2 ± 4.3	-3.3 ± 5.5	0.52
UPDRS-III OFF	31.1 ± 8.8	31.5 ± 12.7	0.78	15.1 ± 9.9	14.2 ± 9.5	-16.0 ± 8.0	-17.3 ± 12.3	0.96
Speech item ON	0.5 ± 0.5	0.3 ± 0.5	0.09	0.1 ± 0.2	0.2 ± 0.3	**-0.4 ± 0.4**	**-0.1 ± 0.5**	**0.03**
Speech item OFF	1.4 ± 0.8	0.9 ± 0.8	0.12	0.4 ± 0.5	0.5 ± 0.6	-1.1 ± 0.7	-0.4 ± 0.9	0.06
Schwab & England (%) ON	**93.8 ± 7.4**	**83.9 ± 9.2**	**0.01**	95.0 ± 5.3	93.9 ± 6.1	**1.2 ± 6.4**	**10.0 ± 10.8**	**0.05**
Schwab & England (%) OFF	71.2 ± 21.7	63.9 ± 21.2	0.21	80.0 ± 14.1	78.3 ± 13.4	8.7 ± 25.3	14.4 ± 21.5	0.36
Hoehn & Yahr ON	1.0 ± 0.8	1.0 ± 0.9	0.95	0.6 ± 1.0	0.8 ± 0.7	-0.4 ± 0.6	-0.2 ± 0.8	0.63
Hoehn & Yahr OFF	2.4 ± 1.0	2.4 ± 0.9	0.80	1.4 ± 5.3	1.6 ± 0.7	-1.1 ± 0.8	-0.9 ± 1.0	0.41
LEDD (mg)	1319.4 ± 767.0	1249.8 ± 459.1	0.78	690.9 ± 603.4	787.8 ± 310.1	-628.5 ± 464.6	-462.0 ± 326.1	0.27
MADRS	5.4 ± 5.6	5.7 ± 4.8	0.48	4.6 ± 5.3	3.6 ± 3.1	-0.7 ± 4.3	-2.1 ± 4.8	0.60
AES	31.6 ± 5.7	31.9 ± 7.7	0.89	35.1 ± 9.7	29.4 ± 6.2	**3.5 ± 5.0**	**-2.4 ± 4.0**	**0.01**
Cognitive items	13.6 ± 2.8	14.2 ± 3.2	0.68	15.7 ± 4.6	13.4 ± 2.9	**2.1 ± 2.4**	**-0.9 ± 1.8**	**0.007**
Behavioural items	8.0 ± 1.2	8.5 ± 2.3	0.61	9.8 ± 2.4	7.6 ± 1.7	**1.8 ± 2.1**	**-0.9 ± 1.9**	**0.009**
Emotional items	4.4 ± 0.5	4.1 ± 1.1	0.39	3.9 ± 1.2	3.5 ± 1.1	-0.5 ± 0.9	-0.6 ± 1.1	0.98
Other items	5.6 ± 1.9	5.2 ± 1.8	0.51	5.8 ± 1.9	4.9 ± 1.6	0.1 ± 1.0	-0.2 ± 1.5	0.56
MDRS								
Without verbal initiation	112.5 ± 1.4	111.3 ± 2.0	0.15	111.6 ± 2.1	111.2 ± 2.2	-0.9 ± 1.6	-0.1 ± 2.9	0.31
Verbal fluency								
Semantic	**38.1 ± 7.3**	**26.5 ± 10.0**	**0.01**	27.1 ± 9.4	29.9 ± 10.5	**-11.0 ± 4.0**	**3.4 ± 3.7**	**<0.0001**
Phonemic	22.5 ± 7.3	21.8 ± 7.4	0.85	20.1 ± 4.8	18.3 ± 7.5	-2.4 ± 4.8	-3.6 ± 4.1	0.72
Stroop								
Colour	75.6 ± 9.9	70.0 ± 16.1	0.27	72.7 ± 8.9	65.6 ± 12.7	-2.9 ± 8.1	-4.4 ± 10.8	0.98
Word	110.5 ± 17.9	97.2 ± 17.6	0.32	104.5 ± 14.6	97.7 ± 17.6	-6.0 ± 9.2	0.4 ± 12.0	0.24
Colour-Word	45.4 ± 21.0	41.2 ± 10.3	0.28	44.4 ± 5.8	40.4 ± 9.2	-1.0 ± 11.1	0.3 ± 5.8	0.62
Interference	0.6 ± 7.5	1.0 ± 7.1	0.77	1.7 ± 4.2	1.3 ± 8.9	1.1 ± 8.4	0.3 ± 5.8	0.82
TMT								
Part A	44.2 ± 21.0	46.3 ± 14.0	0.52	40.9 ± 14.4	47.2 ± 14.5	-3.4 ± 12.0	0.8 ± 9.2	0.29
Part B	107.6 ± 65.2	105.2 ± 49.1	0.72	138.9 ± 97.6	119.7 ± 76.9	31.2 ± 44.7	14.4 ± 41.8	0.44
B-A	63.4 ± 53.0	58.9 ± 40.9	0.85	98.0 ± 84.9	72.5 ± 71.9	34.6 ± 48.4	13.6 ± 44.3	0.34
MCST								
Categories	5.5 ± 1.0	5.6 ± 0.8	0.50	6.0 ± 0.0	5.8 ± 0.3	0.6 ± 1.0	0.2 ± 0.8	0.22
Errors	5.5 ± 3.3	5.4 ± 5.7	0.38	2.5 ± 1.6	4.8 ± 4.1	-3.0 ± 2.5	-0.6 ± 5.4	0.11
Perseverations	1.2 ± 0.9	1.5 ± 2.1	0.76	0.9 ± 1.1	1.6 ± 1.9	-0.4 ± 1.3	0.1 ± 2.6	0.51

*Abbreviations*. *SD* = standard deviation; UPDRS-III = Unified Parkinson’s Disease Rating Scale Part III; LEDD = levodopa equivalent daily dose; MADRS = Montgomery and Asberg Depression Rating Scale; AES = Apathy Evaluation Scale; MDRS = Mattis Dementia Rating Scale; TMT = Trail Making Test; MCST = Modified Wisconsin Card Sorting Test.

^a^Compared with Fisher’s exact test.

### Brain metabolism results

#### Phonemic fluency

**Fig 1:**
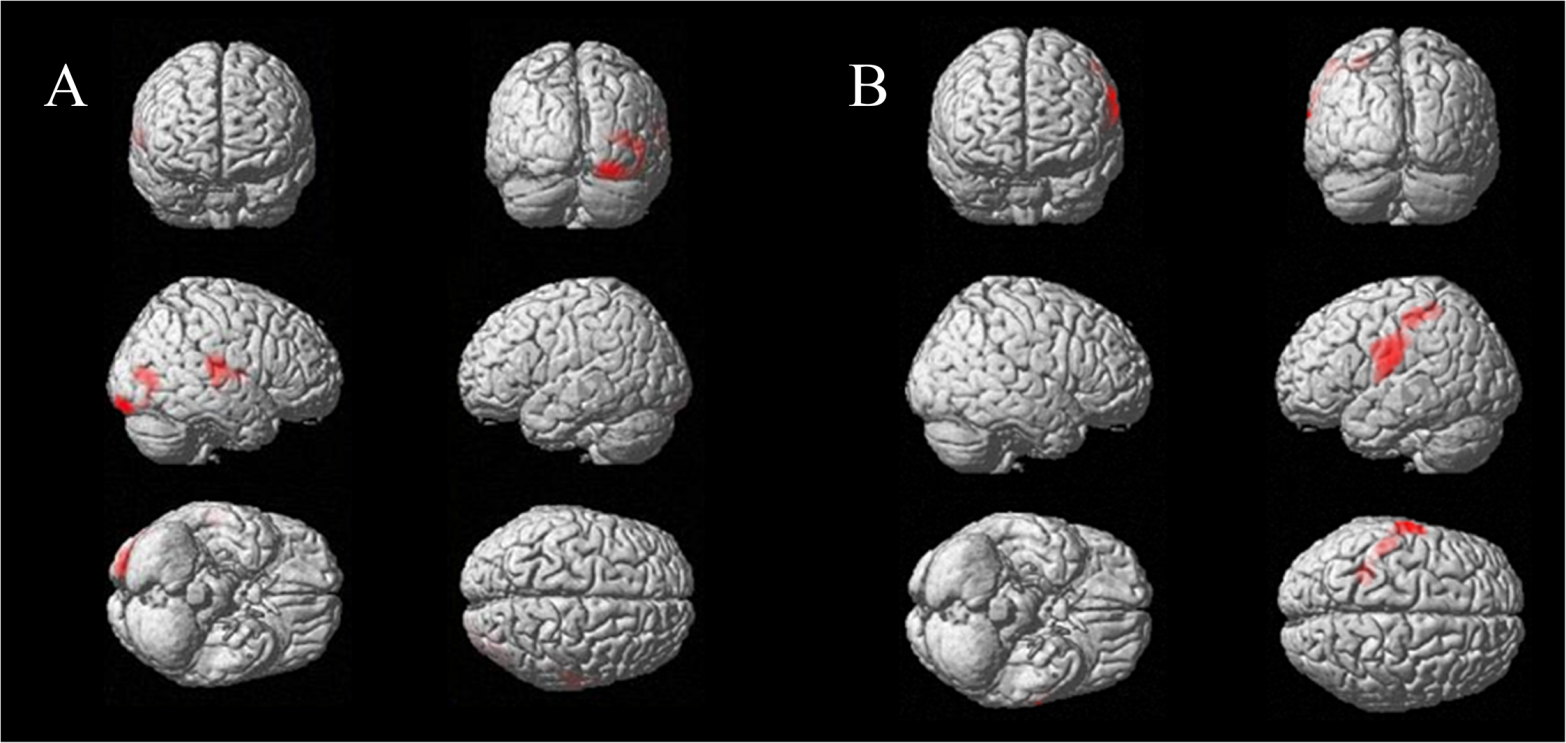
Comparison of changes in brain glucose metabolism between the subgroups (A) with and without impaired phonemic fluency and (B) with and without impaired semantic fluency.

Compared with patients with no phonemic impairment, patients with phonemic impairment exhibited a significant decrease in postoperative metabolism in the right middle occipital gyrus (Brodmann areas (BAs) 19 and 37), right fusiform gyrus (BA 18) and right superior temporal gyrus (BAs 21, 22 and 42) (Table 4, Fig 1A).

**Table 4:** Summary of changes in brain glucose metabolism following STN-DBS (*p* < 0.001, corrected at the cluster level).

Regions	BA	MNI coordinates			Cluster size	*p* value
		X	Y	Z		
***Phonemic fluency***						
Right occipital lobe, fusiform gyrus	18	25	-98	-21	486	0.006
Right occipital lobe, middle occipital gyrus	37	48	-72	-1	486	0.006
Right occipital lobe, middle occipital gyrus	19	34	-82	6	486	0.006
Right temporal lobe, superior temporal gyrus	42	62	-23	12	339	0.023
Right temporal lobe, superior temporal gyrus	22	60	-4	6	339	0.023
Right temporal lobe, superior temporal gyrus	21	53	-23	-1	339	0.023
***Semantic fluency***						
Left frontal lobe, precentral gyrus	43	-62	-7	19	622	0.002
Left parietal lobe, postcentral gyrus	3	-64	-16	28	622	0.002
Left parietal lobe, inferior parietal lobule	40	-29	-44	55	622	0.002

*Abbreviations*. BA = Brodmann area.

#### Semantic fluency

Compared with patients with no semantic impairment, patients with semantic impairment exhibited a significant increase in postoperative metabolism in the left precentral/postcentral gyrus (BAs 3 and 43) and left inferior parietal lobule (BA 40) (Table 4, Fig 1B).

## Discussion

In the present study, featuring a large cohort of patients and an original study design, we investigated the neural networks and behavioural modifications associated with changes in phonemic and semantic verbal fluency in patients with PD following STN-DBS. The first originality of our study is that we divided the STN-DBS cohort into subgroups according to the presence or absence of postoperative changes in either phonemic or semantic verbal fluency (decreased performance or not), comparing performances 3 months before and after surgery. Second, we took the test-retest effect and disease progression into account, by including a group of 34 patients with PD without STN-DBS who were matched at baseline with the STN-DBS group. Third, we compared the subgroups with or without impairment in either phonemic or semantic fluency, adopting a multidimensional approach that encompassed clinical changes in motor, psychiatric and neuropsychological domains, as well as changes in brain metabolism following surgery. This method revealed that (i) the deterioration in these two types of fluency was associated with different neural pathways and behavioural mechanisms, and (ii) there was no evidence to support the hypothesis that a postoperative frontal lobe-related executive dysfunction is responsible for impaired fluency.

The difference in brain metabolism between the subgroups with or without a postoperative deficit in phonemic fluency concerned the right middle occipital gyrus (BAs 19 and 37), right fusiform gyrus (BA 18) and right superior temporal gyrus (BAs 21, 22 and 42). In an fMRI study conducted in healthy participants, the right fusiform gyrus was found to be more activated for phonemic verbal fluency than for semantic verbal fluency, but its role in verbal fluency remains poorly understood [35]. The role of the right middle occipital in verbal fluency has yet to be elucidated. The right superior temporal gyrus has been associated with automatic speech / over-learned associations that could be helpful in verbal fluency [35]. Regarding semantic fluency, we observed a difference between the subgroups in the left inferior precentral/postcentral gyrus (BAs 3 and 43) and left inferior parietal lobule (BA 40). This network is known to be involved in speech production, notably articulatory processes [36]. Thus, different brain areas appear to be involved in postoperative deficits in phonemic or semantic fluency, and even if their role in verbal fluency is not fully understood, none of them are frontal areas involved in cognitive functions.

Schroeder and colleagues (2003) and Kalbe and colleagues (2009) have already described the relationship between decreased phonemic fluency and brain hypometabolism. However, the only change in common in these studies concerned the left inferior frontal gyrus (Broca’s area) [8,9]. The other changes reported in these two studies were observed in the right orbitofrontal cortex [8], left inferior temporal gyrus [8], left dorsolateral cortex [9] and right anterior cingulate cortex [9]. In an ECD-SPECT study, Cilia and colleagues (2007) found that a decrease in semantic fluency was associated with perfusion decreases in the left dorsolateral prefrontal cortex, anterior cingulate cortex and ventral caudate nucleus [10]. The three above-cited studies interpreted reduced phonemic and semantic fluency as mainly reflecting impairment in executive domains. In our study, however, we failed to find any relationship between fluency changes and metabolic changes in frontal areas involved in executive functions. Several hypotheses can be formulated to explain these diverging results. First, the study designs were different. We compared resting-state PET images, whereas previous studies either used a PET activation paradigm, with counterbalanced STN-DBS ON and OFF stimulation states [8], or correlated postoperative changes in verbal fluency with metabolic changes following STN-DBS [9,10]. Second, methods for assessing fluency were different: we used a 2-min version of the phonemic and semantic verbal fluency test, with a single item for each component (i.e., *animal* category and letter *p*), whereas previous studies used shorter and more numerous versions of each fluency assessment. Third, our results were based on the assessment of a large cohort of 26 patients, versus just seven and nine patients in the studies of phonemic fluency [8,9], and 20 patients in the single study of semantic fluency [10]. Fourth, the clinical data published in these studies indicate that our patients were younger and at a less advanced stage of the disease, even if some motor scores were not provided in the three above-cited studies.

Consistent with our brain imaging results, our behavioural data did not support the hypothesis that an executive dysfunction sustained by frontal areas is responsible for the verbal fluency deficit following STN-DBS surgery. An isolated decline in phonemic or semantic verbal fluency, without any decline in other executive functions, has been consistently reported in the literature [11–16]. In line with this, we found a decrease in phonemic fluency but no other executive dysfunction. The only changes we observed concerned not executive processes, but cognitive speed processes. We found a decrease in the Stroop word score when we divided our patient group according to the presence or absence of a postoperative phonemic fluency deficit. Furthermore, the decrease in the Stroop word score and the additional time needed to complete the TMT Part A were significantly correlated with the postoperative decline in phonemic fluency. The fact that the deficit in phonemic fluency was specifically related to a speed reduction in two distinct tasks measuring cognitive speed supports the view of a general cognitive slowdown following STN-DBS, as recently proposed [17,18]. More recently, a reduction in processing speed after surgery has been found to be the primary DBS change [37]. We can therefore speculate that cognitive slowdown is the major DBS side effect, leading to a deterioration in phonemic fluency. It should be noted that this postoperative slowdown seemed not to be related to motor deterioration, as (i) patients exhibiting a phonemic deterioration underwent a greater motor improvement, as measured with the UPDRS III score, and (ii) this motor improvement was not correlated with the change in phonemic fluency.

Our analysis of the change in semantic fluency yielded slightly different findings. We observed that a decrease in semantic fluency was related to an increase in apathy, as assessed with the total AES score and the Cognitive and Behavioural AES subscores. Patients with a postoperative semantic deficit had an increased apathy score: the semantic verbal fluency deficit increased in parallel with the impairment in initiating and sustaining goal-directed behaviour and/or the decrease in goal-related thought. In accordance with the literature, this points to a relationship between the modulation in semantic fluency and the apathy that is ever observed following STN-DBS [14,19], rather than a specific executive dysfunction. In addition, the decrease in semantic fluency seemed not to be related to postoperative speech impairments such as dysarthria or hypophonia, as (i) patients exhibiting semantic deterioration following STN-DBS had a greater speech improvement, as measured with the Speech subscore (Item 18) of the UPDRS III, and (ii) this speech improvement was not correlated with postoperative semantic changes.

This is the first study to have associated functional brain imaging with a broad and multidimensional assessment of neurophysiological, psychiatric and motor functions, with a view to exploring the verbal fluency decrease that can occur following STN-DBS surgery in PD. As a whole, we found no evidence to suggest that this deterioration in verbal fluency results from frontal lobe-related cognitive deficits. First, we found no relationship between the verbal fluency deficit and scores on the neuropsychological tests assessing other executive functions. Second, we found no relationship between this deficit and frontal lobe cognitive areas. Thus, it seems unlikely that dysfunction of frontal lobe-related cognitive processes plays a major role in the semantic or phonemic verbal fluency decrease following STN-DBS. In addition, the changes we observed in some brain areas and the results of the neuropsychological assessment suggest that the phonemic and semantic deficits in fluency are subtended by distinct mechanisms. The deficit in phonemic fluency seems to be associated with a general cognitive slowdown, whereas apathy appears to play a role in the postoperative decrease in semantic fluency.

Nonetheless, several points need to be borne in mind when interpreting our results. Our patients were assessed with neuropsychological tests that are used in standard clinical evaluations in advanced PD. Further studies combining functional imaging and additional behavioural assessments are therefore needed to complete these results. Specifically, the neuropsychological and psychiatric battery should be enlarged to include tests assessing the language domain (e.g., naming, comprehension, object knowledge), other executive functions (e.g., verbal working memory), and other psychiatric changes (e.g., impulse control disorders). Such studies could confirm that the fluency deficit following STN-DBS is unrelated to executive dysfunction sustained by frontal areas, as we suggest in the current study. In addition, more research using functional imaging is needed to further explore the respective roles of apathy and cognitive slowdown in the occurrence of postoperative fluency deficits. We observed a metabolic modification in the postcentral gyrus, as reported by Le Jeune and colleagues (2009) when they assessed apathy in patients with PD undergoing STN DBS [38]. The changes they found, however, were located in the right postcentral gyrus, whereas we found changes in the left postcentral gyrus. A useful method for clarifying the neuroanatomical basis for an apathy-related reduction in verbal fluency following surgery would be to investigate the overlap between changes in the networks subtending verbal fluency and apathy. Finally, there is a need to clarify why only some patients experience a decline in phonemic or semantic fluency, and why only a small proportion of patients develop both deficits. It is important to gain a better understanding of the mechanisms underlying the verbal fluency deficit, as cognitive symptoms impair patients' quality of life following DBS surgery. Investigating these adverse effects using a multidimensional approach is essential, if we are to limit their occurrence and their clinical impact.

## Supporting Information

S1 Table Individual data. (XLSX)

## Abbreviations

PD: Parkinson’s disease
STN: subthalamic nucleus
BA: Brodmann area
STN-DBS: subthalamic nucleus deep brain stimulation
UPDRS: Unified Parkinson’s Disease Rating Scale
AES: Apathy Evaluation Scale
MDRS: Mattis Dementia Rating Scale
^18^F-FDG: 2-deoxy-2[18F]fluoro-D-glucose
MADRS: Montgomery-Åsberg Depression Rating Scale
TMT: Trail Making Test
MCST: Modified Wisconsin Card Sorting Test
